# Neonatal sevoflurane exposure enhances stress-related neurological susceptibility via NKCC1 modulation

**DOI:** 10.1038/s41598-025-18584-9

**Published:** 2025-09-26

**Authors:** Bi-ying Yuan, Li-hui Shan, Jiang Zou, Xiao-hai Xie, Jia-qi Gan, Ben-zhen Chen

**Affiliations:** 1 Department of Anesthesiology, Sichuan Provincial Women’s and Children’s Hospital, The Affiliated Women’s and Children’s Hospital of Chengdu Medical College, Chengdu, Sichuan China; 2 Department of Pathology, Sichuan Provincial Women’s and Children’s Hospital, The Affiliated Women’s and Children’s Hospital of Chengdu Medical College, Chengdu, Sichuan China

**Keywords:** Sevoflurane exposure, Neonatal, Stress, Fear-extinction, Neuron apoptosis, NKCC1, Neuroscience, Physiology

## Abstract

Neonatal exposure to sevoflurane has been associated with neurodevelopmental impairments. However, the mechanisms underlying increased susceptibility to stress-related neurological disorders remain unclear. This study aimed to investigate the brain vulnerability after repeated neonatal sevoflurane exposure in rats to future traumatic stress exposure.P5 male Sprague - Dawley rats were exposed to 3% sevoflurane for 2 h daily from P6 to P8. Bumetanide, an NKCC1 inhibitor, was administered intraperitoneally before sevoflurane exposure. The expression of NKCC1 in the hippocampus at P8 was detected by reverse transcription -polymerase chain reaction and Western blot analysis. The brain vulnerability to stress exposure was assessed at P14-20 by conditioned fear traumatic stress model. The stress response was assessed by measuring serum corticosterone level. Primary hippocampal neurons of neonatal rat were cultured in vitro and exposed to sevoflurane with or without bumetanide pretreatment. The growth activity, intracellular LDH enzyme activity, ROS levels and apoptosis of hippocampal neurons were determined. Repeated neonatal sevoflurane exposure in rats enhanced serum corticosterone secretion after fear conditioning training and induced deficits in fear extinction training and recall.Pretreatment with bumetanide reversed these effects. In cultured primary neonatal rat hippocampal neurons, sevoflurane exposure enhanced the expression of NKCC1, increased the markers of neuronal cytotoxicity and downregulated the expression of Noggin, Drebrin and MAP2. In contrast, pretreatment with NKCC1 inhibitor bumetanide attenuated these adverse effects in vitro. Our results suggest repeated neonatal sevoflurane exposure enhances stress-related neurological susceptibility in rats by upregulating NKCC1, highlighting NKCC1 as a potential therapeutic target for anesthesia-induced neurodevelopmental vulnerability.

**Supplementary Information:**

The online version contains supplementary material available at 10.1038/s41598-025-18584-9.

## Introduction

Neonatal exposure to general anesthetics is associated with long-term neurodevelopmental risks, particularly when repeated during critical windows of brain maturation^1,2^. While single anesthetic exposures may not uniformly induce cognitive deficits in pediatric populations, cumulative evidence links multiple early-life anesthetic or surgical events to heightened susceptibility to learning impairments, behavioral abnormalities, and stress-related neurological disorders^3–5^. Recent reviews further indicate that anesthetic neurotoxicity may correlate with exposure frequency, duration, and individual genetic background, emphasizing the necessity for precision assessment of risk factors^6^. However, the mechanistic interplay between neonatal anesthetic exposure and subsequent environmental stressors remains incompletely understood, limiting translational efforts to mitigate these risks.

Sevoflurane is a cornerstone of pediatric anesthesia, favored for its rapid induction, smooth emergence, and hemodynamic stability. Despite its clinical utility, preclinical and clinical studies have implicated neonatal sevoflurane exposure in neurobehavioral impairments, including anxiety-like behaviors and cognitive deficits^7–9^. Studies in model organisms (e.g.Caenorhabditis elegans) have also confirmed that early developmental exposure to volatile anesthetics induces behavioral defects, providing simplified models for preclinical mechanistic investigations^10^. Notably, similar dysregulation of cation-chloride transporters has been reported with other volatile anesthetics: for example, isoflurane exposure in neonatal mice induces neuroinflammation and imbalances in NKCC1/KCC2 expression, leading to long-term cognitive dysfunction^11^.Notably, the neurodevelopmental consequences of sevoflurane are not solely a direct outcome of anesthetic-induced neuronal injury; rather, they likely reflect altered susceptibility to subsequent environmental challenges. This gap in knowledge—how early anesthetic exposure “primes” the developing brain for stress-related dysfunction—drives the current investigation.A central mechanism underlying sevoflurane’s effects involves gamma-aminobutyric acid type A receptors (GABAA receptors), which regulate synaptic transmission, memory consolidation, and consciousness^12,13^.Dysregulation of the GABA signaling pathway is closely associated with multiple neurodegenerative and developmental disorders, and its dysfunction is considered a core mechanism of abnormal neuroplasticity following anesthesia^14^. GABAA receptors function as ligand-gated chloride ion (Cl^−^) channels, where the direction of chloride ion flux is determined by the gradient between intracellular and extracellular chloride concentrations. During neurodevelopment, neuronal chloride homeostasis is governed by two key transporters: the Na^+^-K^+^-2Cl^−^ cotransporter 1 (NKCC1), which imports chloride ions (Cl^−^), and the K^+^-Cl^−^ cotransporter 2 (KCC2), which exports chloride ions (Cl^−^)^15,16^. In immature neurons, NKCC1 predominates, leading to elevated intracellular chloride; GABAA receptor activation thus induces depolarization, a process critical for neuronal proliferation, migration, and differentiation^17^.Sevoflurane disrupts chloride homeostasis. Cabrera et al. (2020) demonstrated that neonatal sevoflurane exposure dysregulates both NKCC1 and KCC2 in the mouse brain, establishing a direct link between inhaled anesthetics and cation-chloride cotransporter dysfunction^18^.Additionally, Cabrera et al. (2021) explored neonatal anesthesia-induced dysregulation of the epigenome, providing further context for long-term neurodevelopmental impacts^19^.Building on this work, we focus on NKCC1 as a central therapeutic target. We hypothesize that sevoflurane-induced NKCC1 upregulation disrupts neuronal chloride homeostasis. This homeostasis is essential for normal neural proliferation, migration, differentiation, and synaptic function. Disruption of this process impairs developing neural circuits and increases stress-related neurological susceptibility .Furthermore, the NKCC1 inhibitor bumetanide has been shown to restore chloride balance and mitigate neurodevelopmental deficits in preclinical models^20,21^. This evidence supports our proposed mechanism. Salmon et al.^22^ noted that chloride homeostasis-dependent depolarization limits activity-driven formation of glutamatergic synapses in the developing hippocampal circuit, further underscoring the importance of NKCC1-mediated chloride balance in neural development.To address these knowledge gaps, we designed this study with one core hypothesis and two specific objectives. The core hypothesis is that neonatal sevoflurane exposure enhances stress-related neurological susceptibility in rats by upregulating NKCC1, and this adverse effect can be reversed by the NKCC1 inhibitor bumetanide. The two specific objectives are:

In vivo: To verify whether repeated neonatal sevoflurane exposure increases vulnerability to subsequent traumatic stress (using a conditioned fear trauma stress model) in juvenile rats and to evaluate the efficacy of bumetanide intervention;

In vitro: To investigate the impact of sevoflurane on NKCC1 expression and cellular functions (viability, cytotoxicity, ROS production, apoptosis) in primary neonatal rat hippocampal neurons and to assess the protective role of bumetanide.

We tested this hypothesis and objectives using complementary in vivo (male Sprague-Dawley rats) and in vitro (primary hippocampal neurons) experimental models.

## Materials and methods

### Animals and drug administration

The experiment was performed from January 2022 to December 2023.Sprague-Dawley (SD) rats were obtained from the Animal Center of Chengdu Medical College, Chengdu, China. All experimental procedures were conducted in accordance with the International Guidelines for Animal Experiments^23^ and the ARRIVE guidelines^24^. The neonatal rat model used in this study conforms to the classic experimental design for assessing anesthetic neurotoxicity, effectively simulating anesthesia exposure scenarios during the critical period of human neonatal brain development^25^.Neonatal P5 male rats were randomly assigned to three groups: SEV (sevoflurane), SEV + BUM (sevoflurane plus bumetanide), and CON (control), with 48 rats per group. The SEV group was exposed to 3% sevoflurane in O2/N2 (FiO2 50%) for 2 h daily for three consecutive days in a thermostated chamber at 37 ± 1 °C. The CON group was separated from the dams and kept under identical conditions (37 ± 1 °C, FiO2 50%), without sevoflurane exposure. The SEV + BUM group received an intraperitoneal injection of 1.82 mg/kg bumetanide 15 min before daily sevoflurane exposure.

## Experimental design

**Fig. 1:**
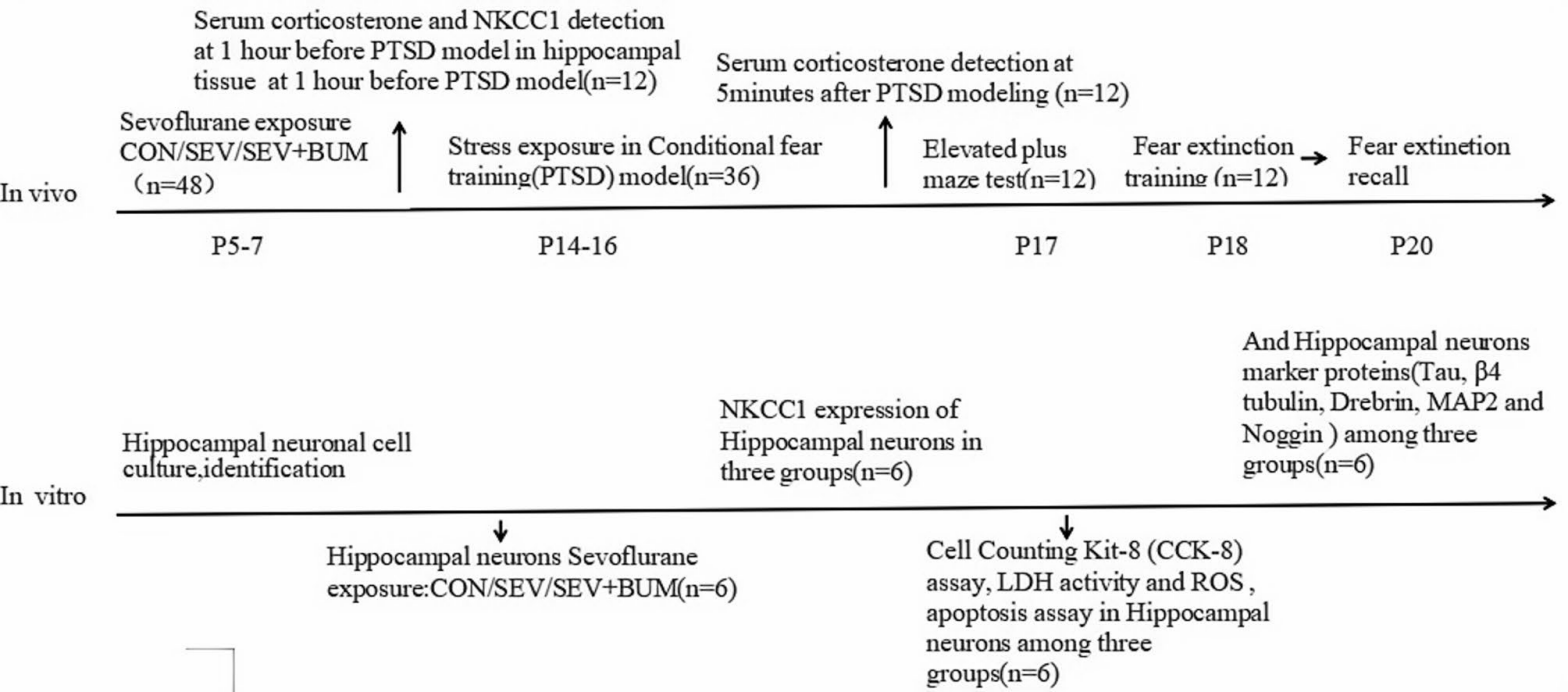
The design roadmap of the article.

The study integrated in vivo (rat model) and in vitro (hippocampal neuronal culture) experiments, structured according to developmental timelines(Fig. 1). In vivo, neonatal male rats (postnatal days 5–7, P5–7) were randomized into three groups (*n* = 48): CON, SEV, and SEV + BUM. At P14–16, rats underwent conditional fear training to induce PTSD-like stress (*n* = 36). Post-stress assessments included serum corticosterone measurement (5 min after PTSD modeling, *n* = 12); anxiety-like behavior evaluation using the Elevated Plus Maze test (P17, *n* = 12); fear extinction training (P18, *n* = 12); and recall testing (P20, *n* = 12). In vitro, primary hippocampal neurons were isolated, cultured, and phenotypically identified. Neurons were assigned to CON, SEV, or SEV + BUM groups (*n* = 6) and exposed to sevoflurane alone (SEV) or sevoflurane combined with bumetanide (SEV + BUM). Post-exposure assays included NKCC1 expression in hippocampal neurons (*n* = 6); cell function analyses including CCK-8 (viability), LDH (membrane damage), ROS, and apoptosis assays. Hippocampal neuronal markers (Tau, β4-tubulin, Drebrin, MAP2, Noggin) were quantified.

## Ethics

Ethical approval for this study was provided by the Ethical Committee of Sichuan Provincial Women’s and Children’s Hospital (Chairperson Prof DeXin-Chen) on 01 March 2021.

## Establishment of the conditioning fear trauma stress (CFTS) model

A fear conditioning system (TSE, Bad Homburg, Germany) was used to establish the conditioning fear trauma stress model at P14. The experimental rats were put into the homemade fear conditioning box and allowed to acclimate to the environment for 5 min. Then, a 4.5 kHz, 60 dB sound signal was presented intermittently 10 times with a 2-minute interval between each sound. The last 5th-10th sound signals were paired with an inescapable foot shock (1.2 mA, 5 s) for 3 days. The appearance of freezing behavior (only breathing and rigid posture of the limbs) in the rats indicated the successful establishment of the conditioning fear trauma stress model. Then, half of the experimental rats were removed for measurement of serum corticosterone, and the other experimental rats were removed from the animal housing. After each fear conditioning session, the fear box was cleaned and wiped with 75% alcohol. Fear extinction training was conducted on the same group of rats on P18. Specifically, following a two-minute adaptation period in the conditioning fear box, the rats received 6 × 2 min of conditioned stimuli (CS), the same sound signal stimuli used for fear conditioning, without paired footshock presentation (with a 2-minute interval). The percentage of rats displaying freezing behavior during each 2 min-CS presentation was recorded to measure fear memory, and freezing behavior was defined as cessation of movement except for respiration, appearing stiff or immobile. Then, the three groups of rats were subjected to 2 min of CS on P20 for the extinction recall test.

## The determination of serum corticosteron

Serum corticosterone levels in blood was measured using a commercial ELISA kit (Cayman.

Chemical Company, Ann Arbor, MI). To investigate the impact of repeated exposures in fear conditioning training on the stress response to foot shocks, serum corticosterone levels were assessed 60 min before fear conditioning training in P14 rats and 5 min after the last unconditioned stimulus (After-US). Blood samples were obtained by the “tail clip” method. the.

distal 0.5 mm of the tail was prescinded and blood was collected directly into microcentrifuge tubes.

### Elevated plus-maze test

The remaining rats in each group (12 rats per group) after fear conditioning training underwent an elevated plus-maze test at P17 to evaluate their anxiety levels. The maze consisted of two open arms (30 cm×5 cm) and two closed arms (30 cm × 5 cm × 15 cm) interconnected by a central platform (5 cm × 5 cm). The maze was constructed of 1 cm thick wooden boards. The maze featured all arms and the central platform in black color and was securely mounted on an adjustable stainless steel frame positioned 40 cm above the ground. At the start of the experiment, the rats were positioned at the center of the maze with their heads facing the open arms. Their activity was recorded and vertically monitored through a video camera. The anxiety level of the rats was assessed by comparing the time spent in the open arms versus the time spent in the closed arms. The percentage of time spent in the open arm was calculated as follows: open arm time percentage = time spent in the open arms/(time spent in the open arms + time spent in the closed arms). Each rat was tested for 5 min.

## Hippocampal neuronal cell culture, identification, and grouping

### Hippocampal neuronal cell culture

Pregnant SD rats at 17–18 days of gestation (purchased from the Experimental Animal Medical College) were subjected to general anesthesia and rapidly dissected to obtain fetal rat hippocampal tissue. The tissue was digested with 0. 125% trypsin at 37 °C for 15 min and centrifuged at 1000 rpm for 10 min to remove the supernatant, and serum was added to stop the reaction. The tissue was washed 3 times with PBS and collected, and a single-cell suspension was made by pipetting 30–50 times. Cells were counted, and the density was adjusted to 6 × 10^6^cells/mL before seeding into 24-well culture plates. Cultures were incubated for 2 h to allow fibroblasts to adhere first, and then the medium containing neurons was transferred to a new plate to continue culturing the purified neurons for 24 h. Then, the serum-containing culture medium was replaced with serum-free culture medium. After 10 days of growth, the expression of the neuron-specific marker NSE in cultured cells was measured by an immunofluorescence assay to verify that the cultured cells were neurons.

### Immunofluorescence

Cell samples were fixed for 20 min at 37 °C in 4% paraformaldehyde (1 ml), washed 3 times with PBS and blocked with 5% normal serum for 1 h at 37 °C. The blocking solution was discarded, and the cells on the slides were incubated overnight at 4 °C with an anti-NSE primary antibody (ab79757, Abcam) diluted 1:150 in 5% BSA. The cells were incubated with secondary antibodies for another 40 min and then counterstained with the nuclear dye DAPI for 20–30 min at 37 °C in a humidified and dark chamber. Cell slides were coverslipped with antifade mountant and imaged on a fluorescence microscope (Olympus). The fluorescence intensity was quantified using ImageJ software (Version 1.53e, National Institutes of Health, Bethesda, MD, USA; URL: https://imagej.nih.gov/ij/).

### Neurons grouping

Hippocampal neuronal cells were randomly divided into 4 groups: (1) the control NC group without any treatment. (2) The sevoflurane group was treated with 4% sevoflurane for 6 h. (3) The bumetanide group was treated with 10 µmol/L bumetanide for 6 h. (4) The sevoflurane + bumetanide group was treated simultaneously with 4% sevoflurane and 10 µmol/L bumetanide for 6 h.

### Cell counting Kit-8 (CCK-8) assay, acid dehydrogenase (LDH) activity and ROS assay

Ten microliters of CCK-8 solution were added to each group of cells, followed by incubation at 37 °C for 1–4 h. The absorbance at 450 nm was measured using a microplate reader. Cell viability was calculated using the following formula: inhibition ratio (%) = [C - B]- [A - B])/[C - B] x 100%, proliferation ratio (%) = [A - B]/[C - B] x 100%, where A is the absorbance of the experimental groups, B is the absorbance of the blank control, and C is the absorbance of the untreated control. The data from three replicate wells were averaged for each group. Moreover, the LDH activity of hippocampal neurons in each group was measured with an acid dehydrogenase (LDH) activity detection kit. A reactive oxygen species (ROS) assay kit was used to assess the intracellular reactive oxygen species (ROS) levels in the four groups of hippocampal neurons.

### Annexin V/PI double-staining combined with flow cytometry was used.

According to the instructions of the apoptosis detection kit, the specific steps were as follows: The hippocampal neuronal cells in the four groups were digested with accutase. To collect the cells, the suspension was centrifuged at 300 × g for 5 min at 4 °C. The collected cells were then washed twice with precooled PBS, resuspended in 1 mL of PBS and centrifuged at 300 × g for 5 min. Then, the cell precipitate was resuspended in 300 µL of binding buffer. Annexin V-FITC (5 µL) was added to the cell suspension, gently mixed and incubated in the dark for 10 min. Then, 5 µL of PI was added to the cell suspension, mixed and incubated in a dark room at 37 °C for 5 min. The stained cells were filtered and analyzed by flow cytometry within 1 h.

### Quantitative real-time PCR

Total RNA from hippocampal neurons in the 3 groups was extracted using TRIzol reagent (Invitrogen, Carlsbad, CA, USA) in accordance with the manufacturer’s instructions, agarose gel electrophoresis was performed, and cDNA was synthesized by reverse transcription. Real-time quantitative PCR was performed according to the instructions of the EnTurbo™ SYBR Green PCR SuperMix kit (ELK Biotechnology, Eq. 001). The primer sequences are shown in Table 1.

**Table 1:** Primer sequences used for quantitative real-time PCR.

Primer name	Primer information	Base sequence (5°–3°)		Tm value	CG%	Product length
R- β-actin	NM_031144.3	Sense	CGTTGACATCCGTAAAGACCTC	59	50	110
		Antisense	TAGGAGCCAGGGCAGTAATCT	58.4	52.4	
R-NKCC1	NM_031798.1	Sense	CGGTTCACGCAGATGACTTG	59.6	55	200
		Antisense	CTTTAGGCGAATGACCACAACT	58.9	45.5	
R-Tau	NM_017212.2	Sense	CCCATGCCAGACCTAAAGAAC	58.9	52.4	152
		Antisense	TGTTTGATATTTGCTTTGAGGC	58.9	39.1	
R-Drebin	NM_031024	Sense	TTCTTCAGACAGCAGGAACGAG	61.3	50.0	177
		Antisense	GATATAGGGAAAGGGAGTCCGT	58.5	50.0	
R-MAP2	NM_013066	Sense	CGGAGTTTAAGATGCAAAGTAA G	56.1	39.0	209
		Antisense	GCTAGGGACTGTTATGCTTGGTTC	59.4	45.8	
R-Tubulin β 4	NM_080882	Sense	AGAGTTAGTGGATGCAGTCCTG	59.5	50.0	167
		Antisense	TATTCATGATCCTGTCTGGGAACT	59.0	41.7	
R-Noggin	NM_012990	Sense	GGCGGCCAGCACTATCTAC	60.3	63.2	115
		Antisense	TCTCGTTCAGATCCTTCTCCTT	58.3	45.5	

Table 1 Detailed information on the primers used for quantitative real-time PCR (qPCR) experiments: Each row includes the primer name, gene information (gene name and accessionnumber), the nucleotide sequence of both the sense and antisense primers, the melting temperature (Tm), the GC content (GC%), and the expected product length (bp). These primers are designed to target specific genes for expression analysis.

### Western blot analysis

Western blot analysis of hippocampal tissue lysates from rats in vivo and cell extracts in vitro from each group were performed using the following primary antibodies: anti-NKCC1 (13884-1-Ap; Peoteintech), anti-Tau (ab76128; Abcam), anti-Drebrin (10260-1-Ap; Peoteintech), MAP2 (ab32454; Abcam), anti-β4 tubulin (ab179509; Abcam), anti-Noggin (ab239520; Abcam) and anti-β-actin (ab8227; Abcam). The secondary antibody was HRP-conjugated goat anti-rabbit IgG (1:2000). The protein bands were analyzed using Quantity One 1-D Analysis Software (Version 4.6.9, Bio-Rad Laboratories, Hercules, CA, USA; URL: https://www.bio-rad.com/en-us/product/quantity-one-1-d-analysis-software).

### Statistical analysis

Normally distributed quantitative datas were presented as mean ± SEM. Single comparisons were performed by independent T tests, and analysis of variance (ANOVA) was followed by Bonferroni post hoc correction for pairwise comparisons or by Kruskal–Wallis nonparametric tests. Statistical analyses were conducted using SPSS 25.0 (IBM Corp, Armonk, NY, USA) and GraphPad Prism 9.0 (GraphPad Software, US). Statistical significance was set at *p* < 0.05.

## Results

### Repeated neonatal sevoflurane exposure increased the mRNA and protein expression of

### NKCC1 in the hippocampus of rat pups, which was mitigated by pretreatment with bumetanide

**Fig. 2:**
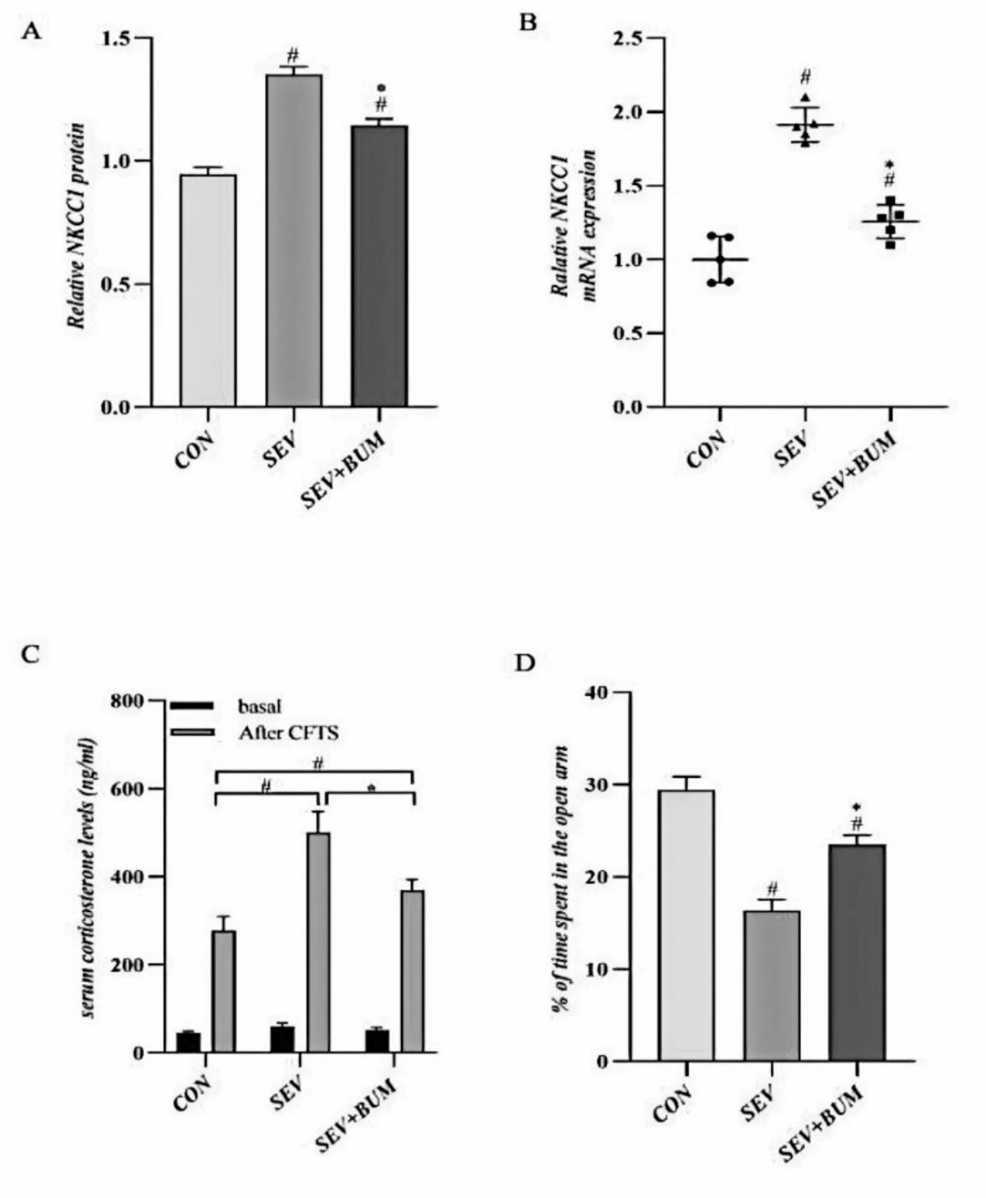
NKCC1 mRNA ( #*p* < 0.0001 vs. CON group; *p < 0.0001 vs. SEV group)and protein expression( #*p* < 0.0001vs CON group; *p = 0.0004 vs. SEV group)were measured in the hippocampus at P14 (**A**, **B**). To evaluate vulnerability to conditioning fear trauma stress (CFTS), the serum corticosterone levels among the three groups were detected at 60 min before and 5 min after the fear conditioning training at P14 (**C**)( #*p* < 0.0001vs CON group; *p < 0.0001 vs. SEV group). Elevated plus maze experiments were conducted on the second day after conditioned fear training. The anxiety level of the rats was assessed by comparing the percentage of time spent in the open arm (**D**) (#*p* = 0.01 vs. CON group; *p = 0.03 vs. SEV group, one-way ANOVA).

Repeated exposure to sevoflurane in neonatal rats led to a significant increase in NKCC1 mRNA (Supplementary Table S1) and protein expression (Supplementary Table S2) in the hippocampus of rat pups at P14. Pretreatment with the NKCC1 inhibitor bumetanide effectively attenuated this upregulation .Specifically, exposure to sevoflurane on postnatal days 6, 7, and 8 resulted in a notable elevation of NKCC1 mRNA and protein levels in the hypothalamic region of neonatal rats. The mitigating effect of bumetanide pretreatment on this increase was statistically significant (*p* < 0.05; Fig. 2A and B). These findings underscore the potential therapeutic efficacy of bumetanide in mitigating the neurobiological consequences associated with early-life exposure to sevoflurane.

### Repeated sevoflurane exposure during the neonatal period increases vulnerability to traumatic stress in SD rats

To determine whether repeated sevoflurane exposure during the neonatal period (P6-P8) affects vulnerability to traumatic stress, the levels of serum corticosterone in rats were detected. Fear conditioning training was administered at P14 for 3 days. Blood samples were collected 60 min before and 5 min after the fear conditioning training. The results showed that there was no significant difference in serum corticosterone levels among the three groups before fear conditioning training. However, 5 min after the fear conditioning training, rats exposed to sevoflurane exhibited significantly higher corticosterone levels compared to the CON group. Pretreatment of neonatal rats with bumetanide significantly attenuated these changes (*p* < 0.05; Fig. 2C). These results indicated that repeated sevoflurane exposure during the neonatal period increased vulnerability to traumatic stress and that bumetanide attenuated the increase in the secretion of corticosterone after fear conditioning training.

### Repeated sevoflurane exposure during the neonatal period increases anxiety levels in the CFTS model

To assess whether there were differences in anxiety levels in SD rats receiving repeated sevoflurane exposure during the neonatal period, elevated plus maze experiments were conducted after conditioned fear training at P17. One-way ANOVA revealed that SEV group spent significantly less time in the open arms than those in the CON group, while this response was significantly alleviated in rats in the SEV + BUM group (*p* < 0.05; Fig. 2D).

### Repeated sevoflurane exposure during the neonatal period in rats led to deficits in fear extinction training and recall

**Fig. 3:**
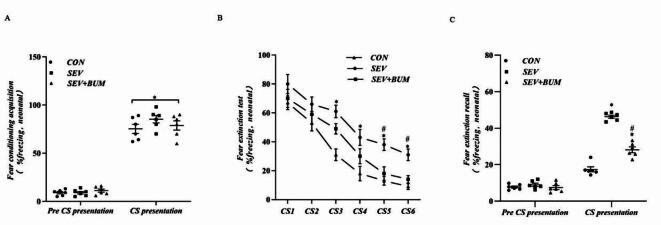
(**A**) Conditioned fear acquisition in the three groups of rats pre-CS presentation among the three groups (*F* = 0.762, *p* = 0.484)and after CS-presentation( F = 1.274,*p* = 0.308) has no significant difference. (**B**) The freezing percentage in fear extinction training in the three groups of rats at CS1 (SEV vs. CON, *P* = 0.1474; SEV + BUM vs. SEV, *P* = 0.2737), CS2(SEV vs. CON, *P* = 0.1359; SEV + BUM vs. SEV, *P* = 0.4442), CS3 (SEV vs. CON, *P* = 0.0035; SEV + BUM vs. SEV, *P* = 0.2028), CS4(SEV vs. CON, *P* = 0.0423; SEV + BUM vs. SEV, *P* = 0.2108),CS5(SEV vs. CON, *P* = 0.0176;SEV + BUM vs. SEV, *P* = 0.0448) and CS6(SEV vs. CON, *P* = 0.0012;SEV + BUM vs. SEV, *P* = 0.0264). (**C**) Fear extinction recall in the three groups of rats pre-CS presentation among the three groups (*F* = 0.858,*p* = 0.44) and after CS-presentation (*p < 0.0001 vs. CON group; #*p* < 0.0001 vs. SEV group ).

This study investigated whether repeated sevoflurane exposure during the neonatal period in SD rats exacerbated deficits in fear extinction training and recall. After repeated sevoflurane exposure during the neonatal period (P6-P8) in SD rats, fear conditioning training was conducted on P14 for three days, and fear conditioning acquisition was performed on P18. There were no significant difference in freezing ratio before CS-presentation (pre-CS presentation) among the three groups (F = 0.762,*p* = 0.484; Fig. 3A). The fear conditioning acquisition among the three groups after CS-presentation has no significant difference ( F = 1.274,*p* = 0.308; Fig. 3A).

Fear extinction training was conducted in the same three groups of rats on P18. Repeated measures analysis of two-way ANOVA indicated a significant difference in the freezing ratio after fear extinction training among the SEV group, CON group, and SEV + BUM groups. Bonferroni post hoc correction revealed that the freezing ratio of the SEV group rats was significantly greater than that of the CON group rats at CS3, CS4, CS5, and CS6, while the freezing ratio of the SEV + BUM group rats was lower than that of the SEV group rats at CS5 and CS6 (*p* < 0.05; Fig. 3B), indicating that the SEV group exhibited a deficit in fear extinction test.

The percentage of rats displaying freezing behavior during each 2 min-CS presentation was recorded to measure fear memory. There was a significant difference in the CS-elicited freezing ratio between CON and SEV group, while the freezing ratio was significantly alleviated in the SEV + BUM group (*p* < 0.05; Fig. 3C), suggesting the impairment in fear extinction recall.These results indicate neonatal exposure to sevoflurane exacerbate fear extinction impairment in rats.

### The cultured cells were verified to be neurons by fluorescence immunocytochemical detection of the neuron-specific marker NSE

**Fig. 4:**
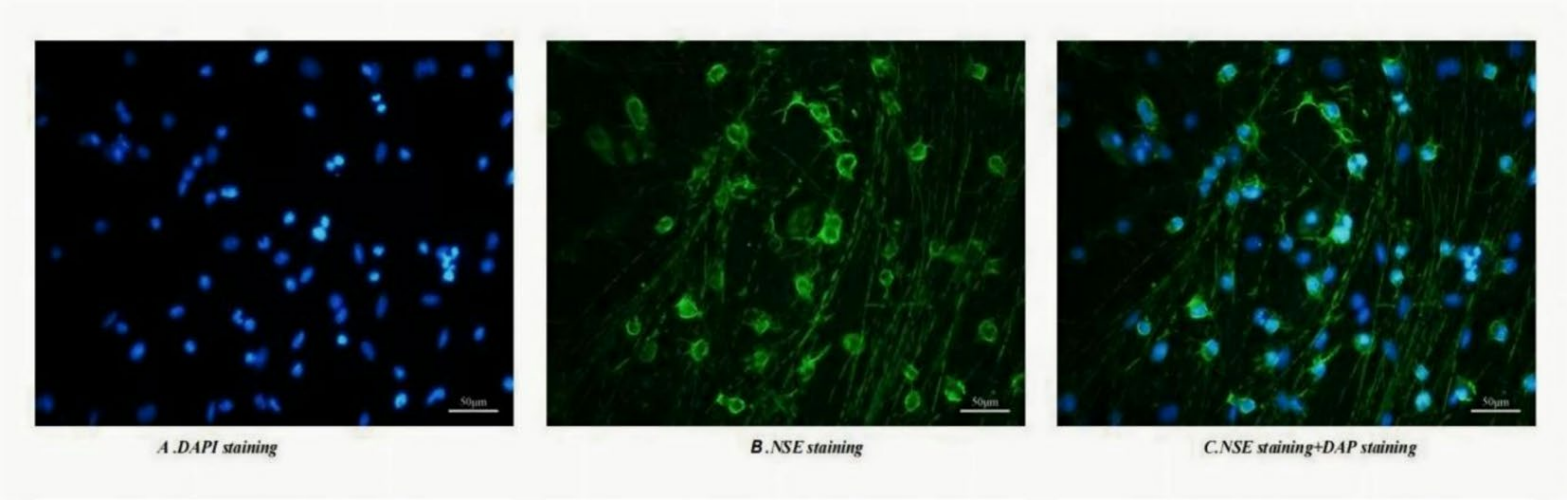
The hippocampal cultured cells were verified to be neurons by fluorescence immunocytochemical detection of the neuron-specific marker NSE. Immunofluorescence staining for NSE (green) and nuclear DAPI (blue) .

Primary cells cultured in vitro were subjected to immunofluorescence staining for NSE, a marker of neuronal cells. Positive immunoreactivity for NSE, visualized in green, was detected in the cytoplasmic compartment of hippocampal neurons (Fig. 4B), whereas cell nuclei counterstained with DAPI fluoresced blue (Fig. 4A). The merged image (Fig. 4C) clearly illustrates the co-localization of NSE (green) and DAPI (blue) within the neurons.These results confirmed the identity of the cultured cells as neurons.

### Exposure to sevoflurane in neuronal cells significantly decreases the viability and enhances LDH activity

**Fig. 5:**
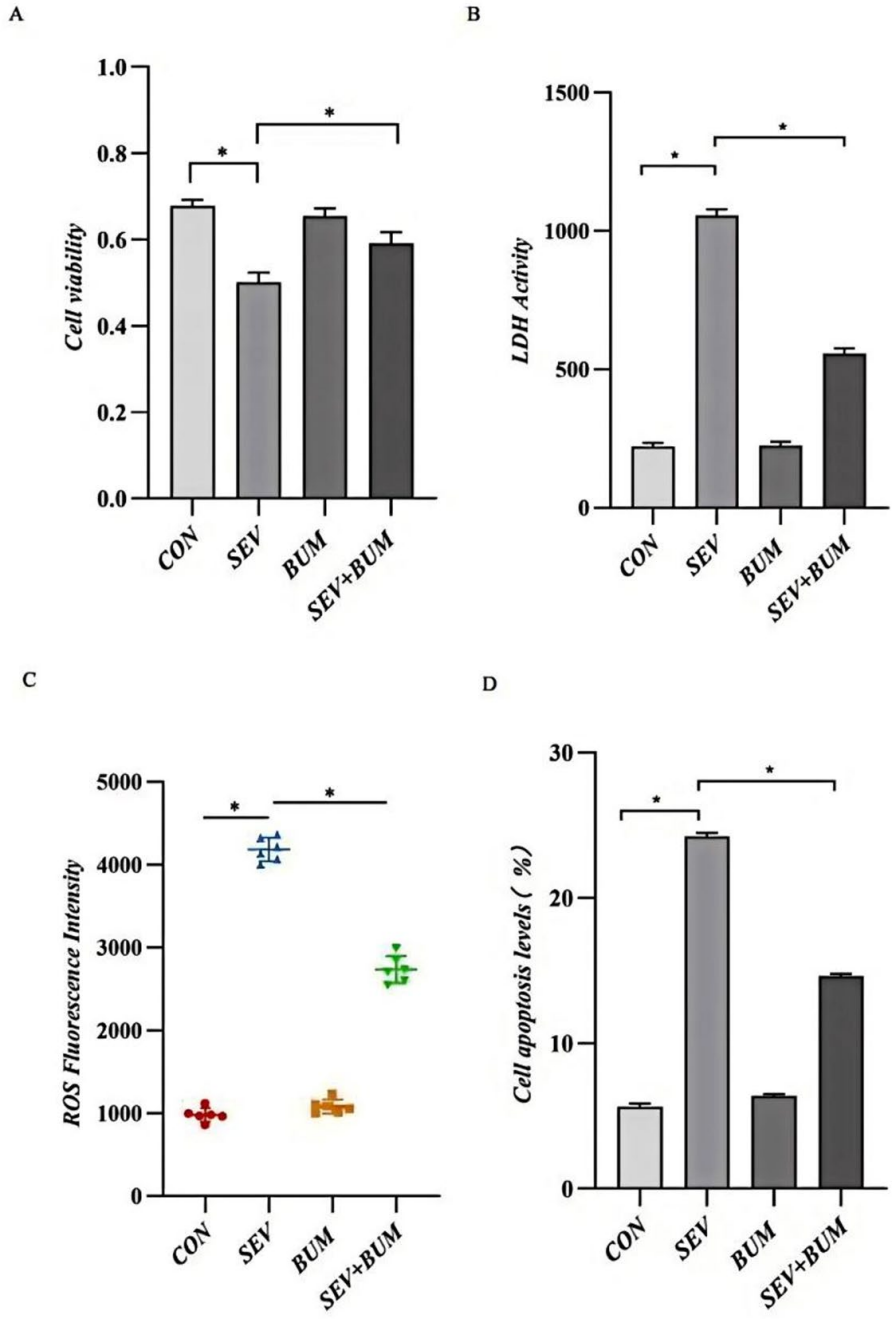
(**A**) CCK-8 assay was used to detect cell viability(SEV vs. CON, *P* < 0.0001;BUM vs. CON, *P* = 0.8345;SEV vs. SEV + BUM *P* = 0.0252), and (**B**)intracellular LDH release was measured as an indicator of membrane damage(SEV vs. CON, *P* < 0.0001;BUM vs. CON, *P* = 0.9985;SEV + BUM vs. SEV, *P* < 0.0001) .(**C**) Intracellular reactive oxygen species (ROS) levels were measured using an ROS detection kit(SEV vs. CON, *P* < 0.0001;BUM vs. CON, *P* = 0.5098;SEV + BUM vs. SEV, *P* < 0.0001). (**D**)Cell apoptosis levels were detected using apoptosis detection kits (SEV vs. CON, *P* < 0.0001;BUM vs. CON, *P* = 0.0630;SEV + BUM vs. SEV, *P* < 0.0001) .

To investigate the effects of sevoflurane on neuronal viability, primary rat hippocampal neurons were cultured and exposed to 4% sevoflurane with or without 10 µM bumetanide pretreatment. Cell viability was quantified by a CCK-8 assay. As shown in Fig. 5A, sevoflurane induced a significant decrease in neuronal viability compared to that in the control group(*p* < 0.01), and this cytotoxicity was markedly attenuated by bumetanide pretreatment (*p* < 0.01). To further assess cytotoxic mechanisms, intracellular LDH release was measured as an indicator of membrane damage. As depicted in Fig. 5B, sevoflurane exposure led to increased LDH release from hippocampal neurons, and this effect was suppressed by bumetanide (*p* < 0.01). Taken together, these data demonstrate that sevoflurane has cytotoxic effects on cultured hippocampal neurons, causing decreased viability and increased membrane damage. Bumetanide appeared to exert protective effects against sevoflurane-induced neuronal cytotoxicity.

### Exposure to sevoflurane in neuronal cells increased oxygen species (ROS) generation

Reactive oxygen species (ROS) play important roles in mediating diverse forms of cellular damage, including proliferation inhibition, senescence, apoptosis and necrosis^26^. In this study, ROS generation in neuronal cells treated with or without bumetanide was evaluated using an ROS assay kit following sevoflurane exposure. Compared with CON group, sevoflurane exposure markedly increased intracellular ROS production in neuronal cells (*p* < 0.0001; Fig. 5C), however, this increase in ROS levels was significantly attenuated by bumetanide cotreatment (*p* < 0.0001; Fig. 5C). Furthermore, sevoflurane exposure also significantly promoted neuronal apoptosis (Fig. 5D), while bumetanide pretreatment reversed this effect (*p* < 0.0001 vs. SEV group) .

### Exposure of neuronal cells to sevoflurane upregulated NKCC1 expression, which was significantly mitigated by pretreatment with bumetanide

**Fig. 6:**
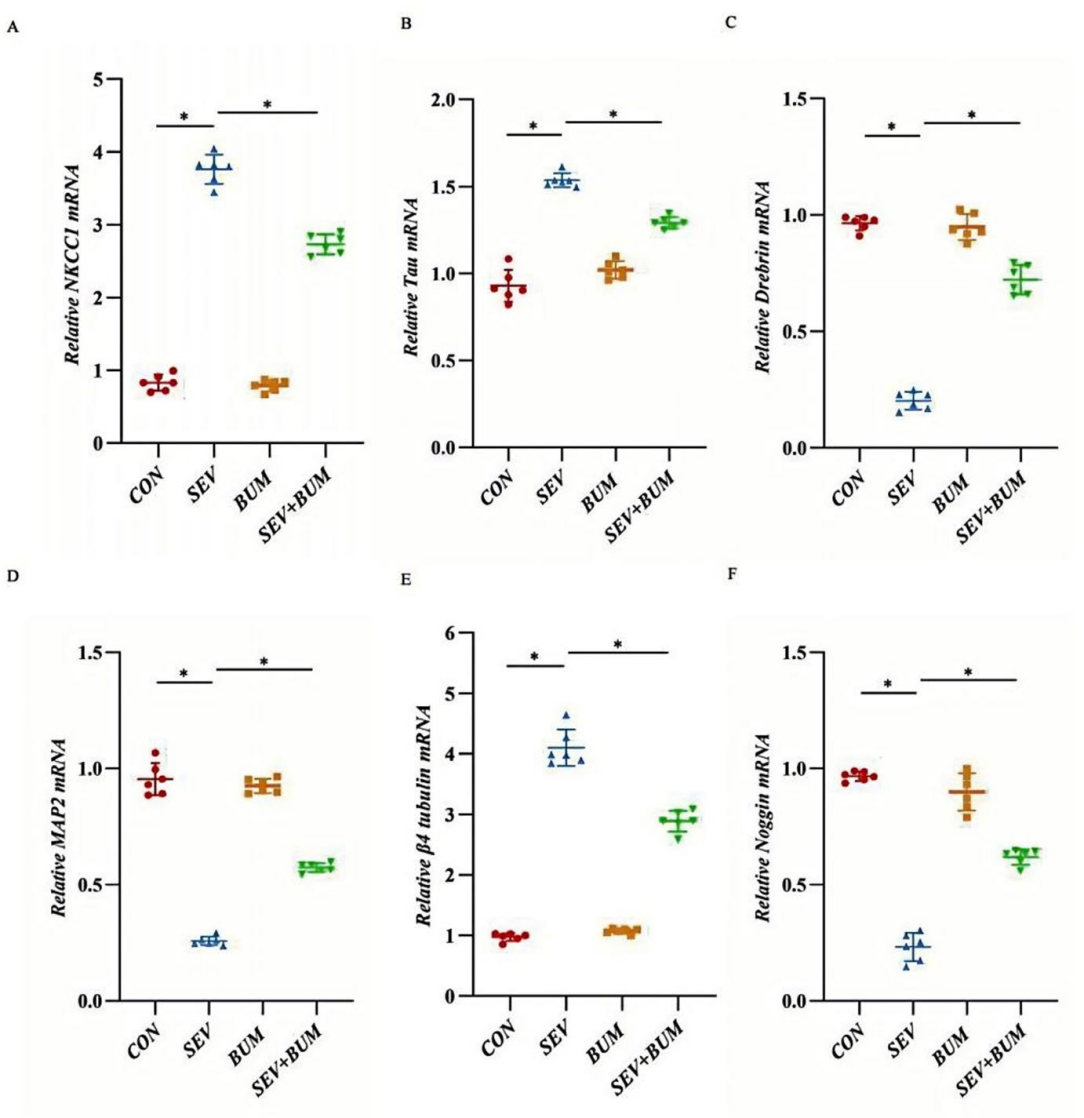
RT‒PCR was used to detect the mRNA expression levels of NKCC1, Tau, β4 tubulin, Drebrin, MAP2 and Noggin for hippocampal cultured Sevoflurane exposure in primary hippocampal neurons increased the mRNA levels of NKCC1 (**A**), tau (**B**) and β4 tubulin (**E**) but decreased the mRNA expression of Drebrin (**C**), MAP2 (**D**)and Noggin (**F**), (mean ± SEM; *n* = 6 wells/group; *p < 0.0001 vs. SEV group; one-way ANOVA).

**Fig. 7:**
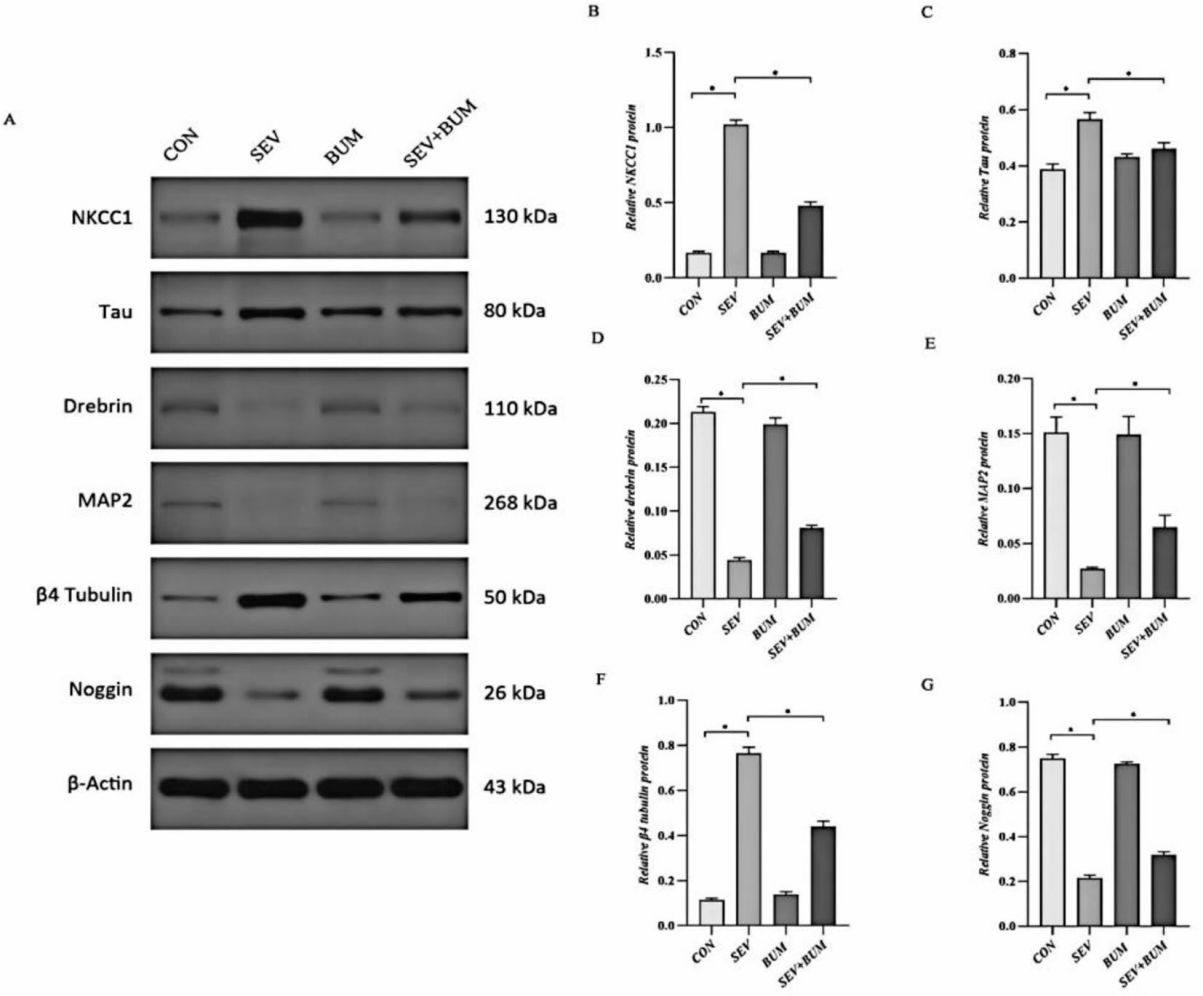
Western blot was used to detect the protein expression levels of NKCC1, Tau, β4 tubulin, drebrin, MAP2 and noggin for hippocampal cultured neurons (**A**–**G**) (mean ± SEM; *n* = 6 wells/group; *p < 0.0001 vs. SEV group; one-way ANOVA).

Our results revealed that sevoflurane exposure led to significant upregulation of mRNA and protein level of NKCC1 in hippocampal neuronal cells, as determined by RT-PCR and Western blot analyses ( *p < 0.0001; Figs. 6A and 7A and B). The NKCC1 inhibitor bumetanide markedly inhibited NKCC1 expression, resulting in notably decreased NKCC1 levels (*p < 0.0001; Figs. 6A and 7A and B), suggesting the involvement of NKCC1 signaling in the effects of sevoflurane.

### Neonatal sevoflurane exposure increased the levels of Tau and β4 tubulin, however, it markedly downregulated noggin, drebrin, and MAP2

Our study demonstrated that sevoflurane exposure altered the expression of several neuronal.

marker proteins related to the cytoskeleton (Tau, β4 tubulin, Drebrin and MAP2) and neurogenesis marker Noggin. The upregulation of Tau and β4 tubulin indicates cytoskeletal damage and impairment of axonal transport (*p* < 0.0001; Fig. 7C and F). Downregulation of Drebrin, MAP2 and Noggin suggested inhibited neurite growth and compromised neuronal differentiation capacity (*p* < 0.0001; Fig. 7D, E and G). Sevoflurane exposure significantly upregulated the mRNA of tau and β4 tubulin and resulted in downregulation of Noggin, Drebrin and MAP2 (*p* < 0.0001; Fig. 6B–F). These changes were associated with sevoflurane-induced neurotoxicity.

Our study revealed that neonatal exposure to sevoflurane can disturb cytoskeletal integrity and inhibit neuronal differentiation in primary hippocampal neurons. Bumetanide regulates intracellular ionic homeostasis and effectively alleviates the cytotoxicity.

## Discussion

Our study demonstrated that repeated postnatal sevoflurane exposure increases stress-related neurological vulnerability in juvenile rats by upregulating the Na^+^-K^+^-2Cl^−^ cotransporter 1 (NKCC1). Bumetanide, an NKCC1 inhibitor, reversed these adverse effects in both in vivo and in vitro models.Below, we discuss mechanistic insights, study limitations, and translational implications.Mechanistic Insights: NKCC1 as a Central Mediator of Sevoflurane-Induced Stress Susceptibility. Bumetanide consistently reduces sevoflurane-induced stress vulnerability. This effect is observed across in vivo measures—such as elevated serum corticosterone, anxiogenic behavior, and fear extinction deficits—and in vitro phenotypes, including neuronal cytotoxicity and cytoskeletal protein dysregulation. These findings identify NKCC1 as a key mediator of sevoflurane-induced neurodevelopmental stress susceptibility. Astrocytic NKCC1 inhibits seizures by buffering Cl^−^^27^, highlighting its broader role in neuron-glial crosstalk and supporting the relevance of NKCC1 dysregulation to neural circuit dysfunction.In developing neurons, NKCC1 is the main transporter that maintains chloride balance, a process essential for normal neuronal proliferation, migration, differentiation, and synaptic function.Our findings demonstrate that sevoflurane exposure upregulates NKCC1 expression in the hippocampus (in vivo, P7) and primary hippocampal neurons (in vitro), while bumetanide (an NKCC1 inhibitor) reverses both NKCC1 overexpression and its associated adverse effects (e.g., increased stress hormone secretion, impaired fear extinction, reduced neuronal viability, elevated ROS production). This confirms that sevoflurane-induced NKCC1 upregulation disrupts chloride homeostasis, leading to impaired neural development and function, which in turn increases susceptibility to stress-related neurological dysfunction.

Chloride homeostasis is closely linked to GABAA receptor-mediated signaling in neural development. For instance, Cabrera et al. (2020) demonstrated that neonatal sevoflurane exposure dysregulates both NKCC1 and KCC2 in the mouse brain. This finding establishes a causal relationship between inhaled anesthetics, cation-chloride cotransporter dysfunction, and GABAergic signaling perturbations.Although our study did not assess GABAA receptor function, KCC2 expression, or other GABA-related molecules, these aspects should be addressed in future research.Future investigations should explore the role of GABAergic neurotransmission by: (1) measuring electrophysiological GABAA receptor-mediated currents, such as mIPSCs in brain slices or primary GABAergic neurons, to directly assess synaptic inhibition; (2) analyzing KCC2 expression to determine whether sevoflurane alters the NKCC1/KCC2 ratio or independently upregulates NKCC1; and (3) employing GABAergic neuron-specific models, including GAD67^+^ sorted neurons or conditional NKCC1 knockout in GABAergic populations, to investigate cell-type-specific contributions to stress-related vulnerability. Despite these novel insights, this work has several limitations that could be addressed by future studies:1 Sex-Specific Considerations Our study exclusively utilized male Sprague-Dawley rats. However, Murguia-Castillo et al. (2013) reported sex-dependent developmental trajectories of NKCC1/KCC2 in the hippocampus and entorhinal cortex. Estrogen-mediated regulation of cation-chloride cotransporters may confer distinct patterns of NKCC1 expression, stress susceptibility, and behavioral phenotypes in female rats post-sevoflurane exposure; this regulation is analogous to the androgenic modulation of NKCC1 in isoflurane neurotoxicity, as shown by Chinn et al. (2020). Thus, our findings are limited to male subjects, and future studies must include female animals to elucidate sex-specific neurodevelopmental responses—critical for translating findings to clinical populations.2 Brain Region Specificity We focused solely on NKCC1 in the hippocampus, yet stress-related neural circuits involve multiple brain regions, including the amygdala (a core structure in fear processing) and prefrontal cortex (involved in stress-related cognitive regulation). Future work should extend NKCC1 analysis to the amygdala and prefrontal cortex to clarify brain-region-specific contributions to sevoflurane-induced stress vulnerability.3 Long-Term Behavioral Persistence Behavioral assessments were confined to the juvenile period (postnatal days 14–20, which approximates the human neonatal-toddler stage). Whether sevoflurane-induced effects persist into adulthood (e.g.; at postnatal day 60 or later) remains unknown. Developmental anesthetic exposure may induce long-term epigenetic reprogramming or neural circuit remodeling(Wang et al. 2008), necessitating longitudinal studies across juvenile, adolescent, and adult stages to determine the temporal dynamics of sevoflurane-induced stress vulnerability.This study identifies NKCC1 as a central mediator of sevoflurane-induced neurodevelopmental stress susceptibility, providing a mechanistic foundation for understanding anesthesia-related neurodevelopmental risks. 新加的4 Due to limitations in experimental conditions, we employed flow cytometry combined with Annexin V/PI staining to detect apoptosis in neurons. Since primary neurons are adherent, we used Accutase for gentle digestion to minimize mechanical damage before quantifying apoptotic cells via Annexin V/PI staining and flow cytometry. This approach allowed high-throughput detection and improved the reliability of apoptosis assessment, but flow cytometry-based detection of adherent neurons still poses technical challenges. Future studies could optimize experimental design by employing live-cell imaging. This approach can overcome flow cytometry’s limitations, including artifacts caused by mechanical damage or handling, as well as the inability to track apoptosis dynamically.Clinically, NKCC1 inhibitors (e.g.: bumetanide) may emerge as a novel neuroprotective strategie for infants at high risk of repeated anesthetic exposure.

## Conclusion

Our results suggested repeated sevoflurane exposure in neonatal rats might increase brain vulnerability to future stress exposure by increasing the expression of NKCC1.

## Supplementary Information

Below is the link to the electronic supplementary material.

Supplementary Material 1

Supplementary Material 2

Supplementary Material 3

Supplementary Material 4

## Data Availability

The original contributions presented in the study are included in the article. Further inquiries can be directed to the corresponding author.
