# Supplementary material for: Neonatal sevoflurane exposure enhances stress-related neurological susceptibility via NKCC1 modulation
Source: Sci Rep. 2025 Sep 26;15:33150. doi: 10.1038/s41598-025-18584-9 (PMC12474865; doi:10.1038/s41598-025-18584-9)
Supplement: Supplementary file 1 — Supplementary Material 1 [file 41598_2025_18584_MOESM1_ESM.pdf]

sample		the grayscale value		
1		NKCC1	β-Actin	NKCC1/β-Actin
	CON	626664	755017	0.83
	SEV	959218	743580	1.29
	SEV+BUM	866099	759736	1.14
2	CON	627100	667128	0.94
	SEV	817953	654075	1.25
	SEV+BUM	761473	650832	1.17
3	CON	690000	750000	0.92
	SEV	999000	740000	1.35
	SEV+BUM	836000	760000	1.10
4	CON	739900	755000	0.98
	SEV	1050450	745000	1.41
	SEV+BUM	826200	76500	1.08
5	CON	798000	759800	1.05
	SEV	1095000	750020	1.46
	SEV+BUM	970200	770000	1.26
6	CON	720100	758010	0.95
	SEV	1009800	748232	1.35
	SEV+BUM	853440	762012	1.12
7	CON	666,100	748,500	0.89
	SEV	978,300	746,800	1.31
	SEV+BUM	846,900	748,900	1.131
8	CON	696,400	748,800	0.93
	SEV	1,000,400	752,200	1.33
	SEV+BUM	864,600	751,800	1.15
9	CON	720,800	751,000	0.96
	SEV	1,024,300	753,200	1.36
	SEV+BUM	873,400	753,000	1.16
10	CON	730,200	752,800	0.97
	SEV	1,041,500	755,100	1.38
	SEV+BUM	891,000	755,100	1.18
11	CON	744,800	752,300	0.99
	SEV	1,048,100	754,000	1.39
	SEV+BUM	899,300	755,700	1.19
12	CON	753,000	753,000	1
	SEV	1,066,800	757,000	1.41
	SEV+BUM	909,600	758,000	1.2

	NKCC1/β-Actin
CON	0.945±0.066
SEV	1.352±0.070

SEV+BUM 1.145±0.059
