# Supplementary material for: Neonatal sevoflurane exposure enhances stress-related neurological susceptibility via NKCC1 modulation
Source: Sci Rep. 2025 Sep 26;15:33150. doi: 10.1038/s41598-025-18584-9 (PMC12474865; doi:10.1038/s41598-025-18584-9)
Supplement: Supplementary file 3 — Supplementary Material 3 [file 41598_2025_18584_MOESM3_ESM.doc]

**Legend for supplementary Table S2: Relative gray value statistics of WB bands for NKCC1 protein expression.**

This table shows the quantitative analysis results of NKCC1 protein and internal reference protein (β- Actin) bands in each experimental group，as well as the ratio of NKCC1/β- Actin, across different samples (numbered 1–12) and experimental groups. Data are presented as mean±SD from 3 independent experiments.

Sample: Indicates the sample number.

Groups:

-CON: Control group.

- SEV: Group treated with sevoflurane.

- SEV + BUM: Group treated with sevoflurane combined with bumetanide.

-NKCC1: Grayscale value of the NKCC1 protein band.

-β- Actin: Grayscale value of the β- Actin protein band (used as an internal reference).

- NKCC1/β- Actin: Ratio of NKCC1 grayscale value toβ- Actin grayscale value, reflecting the relative expression level of NKCC1 normalized to the internal reference.
