# Supplementary material for: Neonatal sevoflurane exposure enhances stress-related neurological susceptibility via NKCC1 modulation
Source: Sci Rep. 2025 Sep 26;15:33150. doi: 10.1038/s41598-025-18584-9 (PMC12474865; doi:10.1038/s41598-025-18584-9)
Supplement: Supplementary file 4 — Supplementary Material 4 [file 41598_2025_18584_MOESM4_ESM.doc]

**Legend for Supplementary Table S1**

This table presents the relative expression levels of NKCC1 mRNA normalized to the internal reference geneβ- Actin, calculated using the 2^{-\triangle\triangle Ct} method. Values represent the fold change in NKCC1 mRNA expression across three experimental groups:

-CON: Control group.

- SEV: Group treated with sevoflurane.

- SEV + BUM: Group treated with sevoflurane combined with bumetanide.

Data in the first section show individual sample values for each group (n = 12 per group), while the second section reports mean±SD for each group.
